# Urokinase-based lock solutions for catheter salvage: A case of an upcoming kidney transplant recipient

**DOI:** 10.1515/med-2023-0699

**Published:** 2023-04-13

**Authors:** Cong Xia, Junfen Fan, Chao Xu, Shouci Hu, Hongzhen Ma, Lingzhi He, Liqing Ye

**Affiliations:** Department of Nephrology, The First Affiliated Hospital of Zhejiang Chinese Medical University (Zhejiang Provincial Hospital of Chinese Medicine), Hangzhou, China; Department of Nephrology, The First Affiliated Hospital of Zhejiang Chinese Medical University (Zhejiang Provincial Hospital of Chinese Medicine), No. 54 You-Dian Road, Hangzhou, China

**Keywords:** catheter-related bloodstream infection, antibiotic lock, urokinase, compatibility, case report

## Abstract

Catheter-related bloodstream infection (CRBSI) is a significant complication among patients on haemodialysis (HD) who are dependent on a central venous catheter (CVC) for an extended period. Catheter removal as first-line treatment can induce accelerated venous access site depletion in patients on HD who rely on it to survive. It is possible to retain the catheter in stable patients without septic syndrome while administering systemic antibiotics and antibiotic lock therapy. Herein, we report the case of a patient on HD with CRBSI who was successfully treated with intravenous levofloxacin- and urokinase-based antibiotic lock, without catheter removal prior to kidney transplantation. The use of urokinase in combination with antibiotics in lock solutions for treating catheter infections is rare. We verified the physical compatibility of levofloxacin and urokinase by visual inspection, turbidimetric measurements, and particle count. To our knowledge, this was a rare case demonstrating the effective use of urokinase and levofloxacin in a catheter lock for CRBSI in a patient on HD. Considering the need for highly concentrated antimicrobials and the availability of various antibiotics, the compatibility and stability of the lock solution is a matter of concern. Further studies are warranted to assess the stability and compatibility of various antibiotics in combination with urokinase.

## 1 Introduction

Catheter-related bloodstream infection (CRBSI) is a common complication of haemodialysis (HD) catheter use [1]. Several treatment strategies for CRBSI have been explored, including antibiotic administration while retaining the catheter or replacing the catheter over a guidewire [2]. Catheter management strategies for CRBSI, particularly in patients on HD, have been controversial [3]. Catheter salvage is essential in HD-dependent patients with limited options for alternate access sites. When a central venous catheter (CVC) needs to be retained, antimicrobial lock therapy in conjunction with systemic antimicrobials is used for treating CRBSI [4], which can benefit clinically stable patients with low-virulence bacteraemia [5].

Most lock trials involve heparin and antimicrobial agents, some of which have been proven incompatible for long periods [6]. Urokinase is an effective thrombolytic agent used to restore CVC patency [7]; however, it is rarely used for catheter infections. To date, no study has evaluated the compatibility and stability of urokinase and antimicrobial agents. Here, we describe our attempt to treat a patient with CVC infection with urokinase-based antibiotic lock therapy without catheter removal. In addition, we verified the compatibility between urokinase and antibiotics.

## 2 Case description

A 32-year-old woman with chronic kidney disease secondary to chronic glomerulonephritis had been on stable maintenance HD for approximately 9 months. The vascular access was a tunnelled catheter placed 8 months prior via the right internal jugular vein. The patient had refused arteriovenous fistula creation because she had been waitlisted for kidney transplantation. She had a history of hypertension and glomerular nephritis. In July 2020, after 2 h of HD, she developed nausea and rigor, with a temperature of 38.4°C. Her blood pressure decreased from 140/89 to 112/65 mmHg and her heart rate was 90 beats/min (regular). Two sets of specimens were obtained for blood culture from the catheter lumen and peripheral vein.

The patient was then admitted to the hospital for further observation. Physical examination revealed no signs of hyperaemia, tenderness, drainage from the exit site, or subcutaneous tunnel of the catheter. Auscultation of her chest and abdominal examination were normal. No obvious causes of non-catheter-related infection were observed. A complete blood count at the time of admission revealed a slightly increased absolute neutrophil count (8.5 × 10^9^ cells/L) with a dramatic increase in the neutrophil/lymphocyte ratio. Her haemoglobin level was 10.6 g/dL and her C-reactive protein was 6.6 mg/L. Echocardiography, chest computed tomography, and abdominal ultrasonography were performed; however, no source of infection was revealed. Therefore, empirical treatment with oral cefuroxime axetil (500 mg/day) was initiated for presumed CRBSI.

On the patient’s third day of hospitalisation, blood cultures from the catheter lumen and peripheral vein revealed *Acinetobacter baumannii*, with sensitivity to ceftazidime, cefoperazone/sulbactam, ciprofloxacin, levofloxacin, piperacillin/tazobactam, ticarcillin/clavulanate, meropenem, imipenem, and sulfamethoxazole. However, she developed a positive reaction in the skin test for ceftazidime and cefoperazone/sulbactam. Therefore, we chose 500 mg of intravenous levofloxacin (quinolone) to be administered every other day, which was tailored to the residual renal function. Antibiotic lock therapy was used to salvage the catheter. The levofloxacin (2 mg/mL) and heparin (1,000 U/mL) mixture produced turbidity. Therefore, we prescribed an antimicrobial lock of levofloxacin (2 mg/mL) and urokinase (5,000 U/mL) once daily.

**Figure 1:**
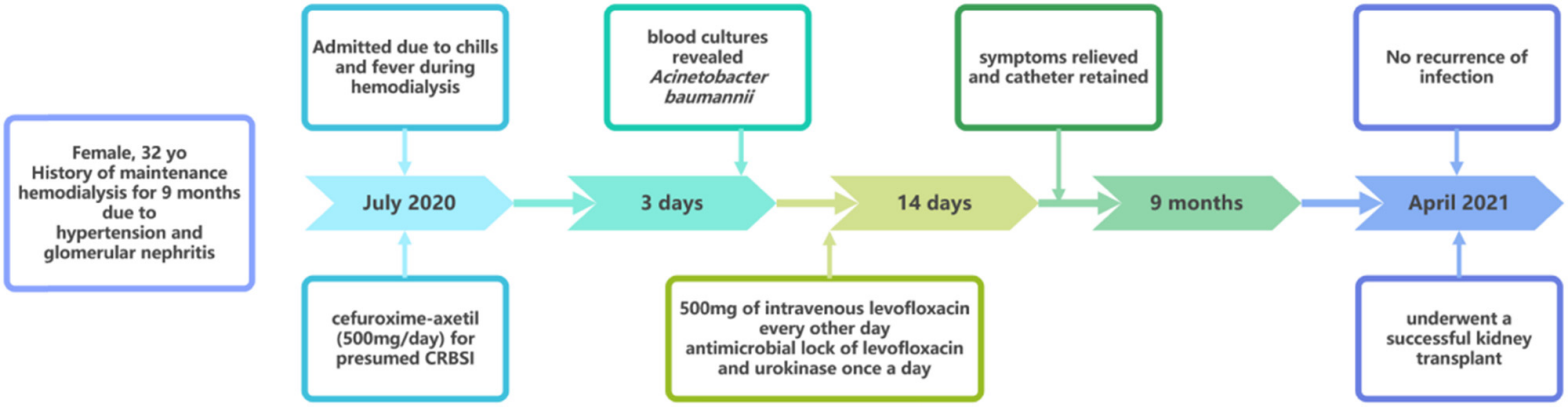
Event timeline of the case.


The patient’s symptoms resolved after the initiation of appropriate antibiotics. Subsequent blood cultures obtained 3 days after treatment initiation was negative; thus, the catheter was salvaged. The effects of treatment on the routine laboratory parameters are shown in Table 1. Intravenous antibiotic treatment and antibiotic lock therapy were administered for 14 days, without any allergic reactions. The patient did not develop a recurrence of infection over the next 9 months, after which she underwent successful kidney transplantation (Figure 1). Additionally, we verified the physical compatibility of levofloxacin and urokinase by visual inspection, turbidimetric measurements, and particle count.

**Table 1:** Biochemical parameters of the patient before and after treatment

Laboratory markers	Before	After	Reference range	Unit
Blood urea nitrogen	19.5	23.7	2.6–7.5	mmoI/L
Creatinine	886	1,032	45–84	µmoI/L
Sodium	142.9	138.2	137–147	mmol/L
Potassium	3.93	5.59	3.5–5.3	mmol/L
Total protein	69.9	61.9	65–85	g/L
Albumin	38.9	35.6	40–55	g/L
Total bilirubin	7.6	7.5	3.4–20.5	µmoI/L
Direct bilirubin	2.7	2.7	0–8.6	µmoI/L
Alkaline phosphatase	59	47	35–100	U/L
Aspartate aminotransferase	5	12	13–35	U/L
Alanine aminotransferase	8	8	7–40	U/L
Gamma glutamyl transferase	8	6	7–45	U/L
Procalcitonin	2.11	0.19	0–0.046	ng/mL
C-reactive protein	13.6	0.2	1–8	mg/L
Haemoglobin	9.8	9.3	11.5–15.0	g/dL
Platelet count	172 × 10^9^	149 × 10^9^	125–350	1/L
White blood cell count	7.3 × 10^9^	4.3 × 10^9^	3.5–9.5	1/L

**Informed consent:** Informed consent has been obtained from patient included in this study.

## 3 Discussion

Infection is the second leading cause of death in individuals with end-stage renal disease (ESRD), who are particularly vulnerable to bloodstream infections [1]. In comparison to arteriovenous fistulae or grafts, catheters are associated with an increase in the rate of infection [8]. Despite CVCs being the primary source of bacteraemia, they are widely used in patients when initiating HD. In the current China Dialysis Outcomes and Practice Pattern Study Phase 5 (DOPPS5) analysis, 11.8% of the prevalent dialysis patients had CVCs *in situ* for access [9].

When considering modalities for the management of CRBSI, the need to preserve venous access is a key consideration for patients with ESRD. According to the guidelines of the Kidney Disease Outcome and Quality Initiative (KDOQI) and the Infectious Diseases Society of America (IDSA), patients with uncomplicated CRBSI caused by Gram-negative bacilli can be initiated on empirical systemic antibiotics and antibiotic lock therapy without immediate removal of the catheter, allowing the salvage of the catheter [10,11]. After 72 h, if symptoms have resolved and there is no sign of metastatic infection, the catheter may be reserved. The causative agent in our case was a non-resistant strain of *A. baumannii*. We attempted to retain the catheter without creating new access, considering the patient’s requirements in preparation for kidney transplantation.

**Figure 2:**
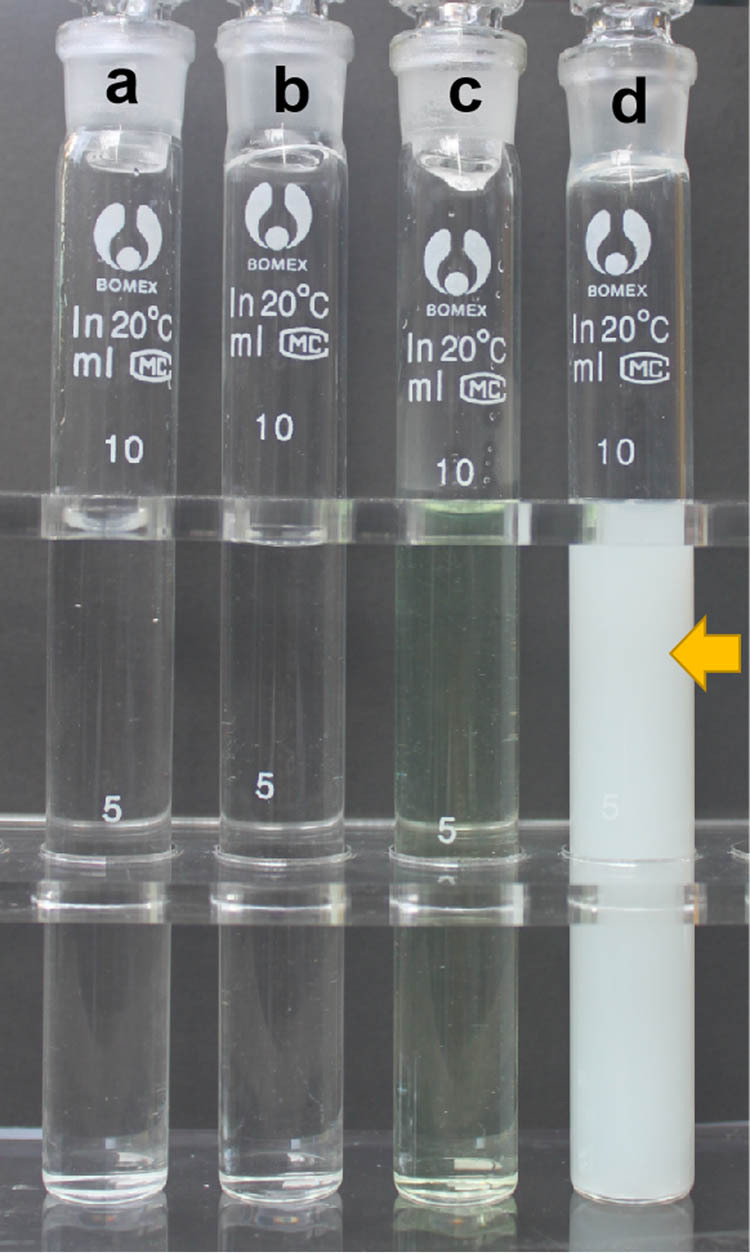
Visual inspection of (a) 0.9% sodium chloride solution, (b) heparin (1,000 units/mL), (c) levofloxacin (2 mg/mL), and (d) a mixture of heparin (1,000 units/mL) and levofloxacin (2 mg/mL). The precipitation is marked with an arrowhead.

Antibiotic lock solutions typically used to eradicate biofilms contain anticoagulants. Heparin instillation is still commonly used to prevent clotting of the catheter-lock solution. However, its antibacterial effects are debated. Heparin reportedly promotes biofilm formation by *Staphylococcus aureus* [12]. Furthermore, Capdevila et al. discovered that heparin does not exert antibacterial action against *S. aureus* [13]. In view of the patient’s positive reaction to the skin test for cephalosporins, fluoroquinolones were chosen as antibiotic agents. Levofloxacin (2 mg/mL) and heparin (1,000 U/mL) were mixed and macroscopic precipitation was observed, as previously described (Figure 2).

Urokinase is a thrombolytic enzyme found in urine that converts plasminogen to active plasmin, lyses fibrin, and subsequently, causes the disintegration of fibrin clots. Thus, urokinase can lyse and expose bacteria nesting within the fibrin sheath [14]. Urokinase, when used as a lock solution for patients with intravascular devices, may reduce the risk of bloodstream infections [15]. Furthermore, the intrinsic antibacterial activity of urokinase may be linked to the modulation of innate and adaptive immune responses [16]. Wang et al. proved that urokinase and a sensitive antibiotic efficiently eliminate coagulase-negative *Staphylococcus epidermidis* embedded in biofilms *in vitro*. For patients on HD with CRBSI, a urokinase-based antibiotic lock is more effective than a heparin-based lock [17].

**Figure 3:**
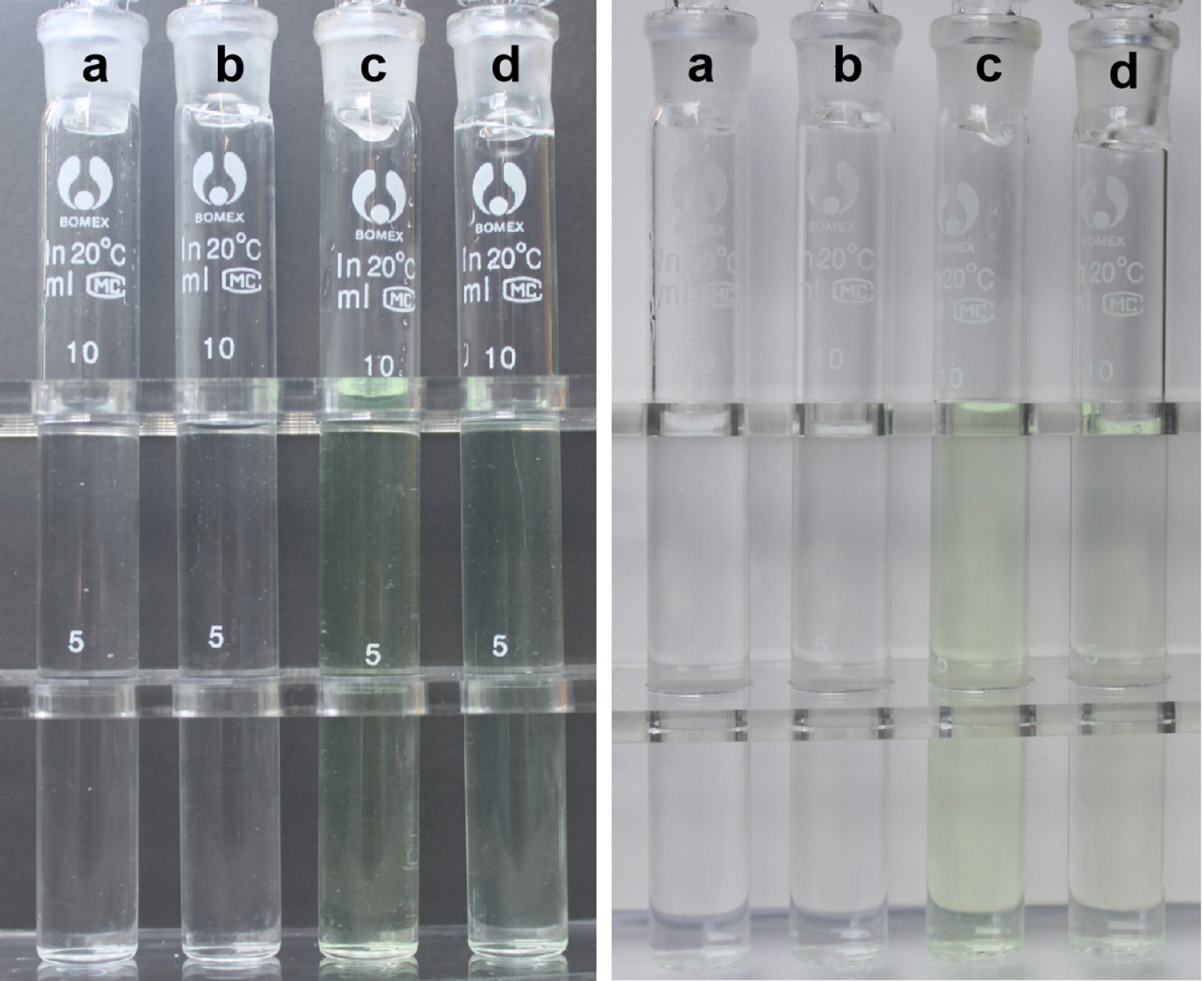
Visual inspection of (a) 0.9% sodium chloride solution, (b) urokinase (5,000 units/mL), (c) levofloxacin (2 mg/mL), and (d) a mixture of urokinase (5,000 units/mL) and levofloxacin (2 mg/mL) against a black and white background immediately after preparation.

**Figure 4:**
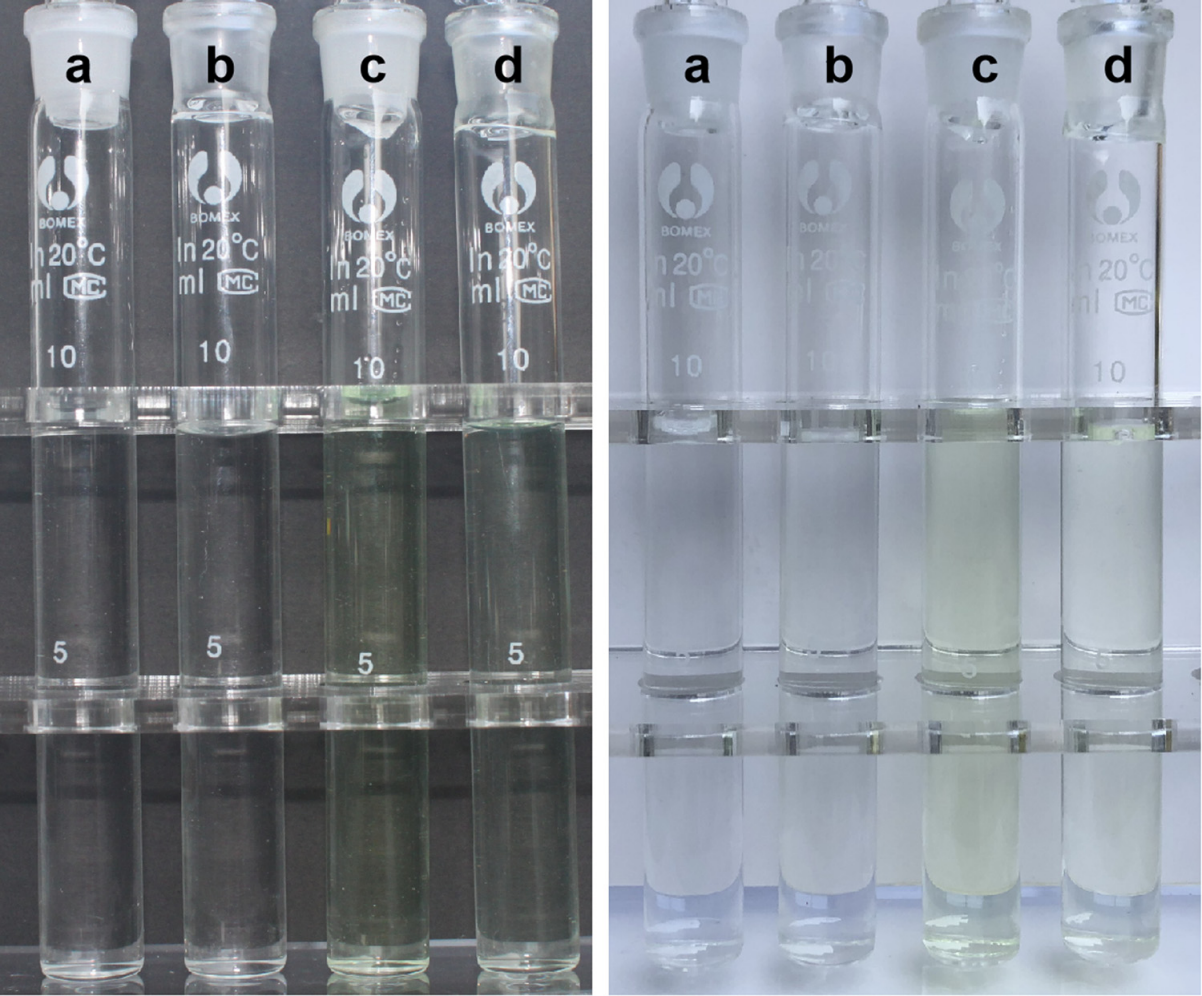
Visual inspection of (a) 0.9% sodium chloride injection, (b) urokinase (5,000 units/mL), (c) levofloxacin (2 mg/mL), and (d) a mixture of urokinase (5,000 units/mL) and levofloxacin (2 mg/mL) against a black and white background after 24 h.

Urokinase has rarely been used in combination with antibiotics in lock solutions for catheter infections. To ensure the removal of bacteria within the biofilm, antimicrobial concentrations within the catheter need to be 100 times greater than the minimum inhibitory concentration [4], which may affect its compatibility with urokinase. In our case, the minimum effective concentration of urokinase reported to be effective in restoring catheter patency [18] was chosen to prevent incompatibility. Furthermore, this case demonstrated the compatibility of levofloxacin and urokinase. A mixture of levofloxacin (2 mg/mL) and urokinase (5,000 U/mL) at room temperature showed no turbidity, colour change, visible particles, or gas formation under normal room lighting in a sterile, clear, colourless glass tube against a black or white background after preparation or after 24 h (Figures 3 and 4). Thereafter, the mixture was analysed for non-visible changes using a laboratory turbidimeter and for subvisual particles using a particle counter. Physical incompatibilities were defined as changes in turbidity of ≥0.5 nephelometric turbidity units (NTUs) [19]. The absolute turbidity difference was <0.5 NTU between the test and control groups at different time points (Table 2). According to the European Pharmacopoeia 10.0, ≥10 µm-sized particles at a concentration of ≤25 particles/mL and ≥25 µm-sized particles at a concentration of ≤3 particles/mL indicate compatibility [20]. Immediately after mixing and 24 h later, no particles were greater than 3 µm. Therefore, it can be definitively said that 2 mg/mL levofloxacin is physically compatible with 5,000 units/mL urokinase within a period of 24 h.

**Table 2:** Turbidity difference between the test and the control group^a^

Drug	Time after admixing	
	0 h	24 h
Levofloxacin (2 mg/mL) Urokinase (5,000 U/mL)	Increase of 0.42 NTU^b^	Increase of 0.48 NTU

^a^Control group was levofloxacin (2 mg/mL); ^b^NTU: nephelometric turbidity unit.

## 4 Conclusion

In summary, this was the case of a 32-year-old female with CRBSI who was on the waiting list for kidney transplantation. She was successfully treated with systemic antibiotics in combination with a urokinase-based antibiotic lock solution, without catheter removal. In patients undergoing HD, a tailored approach is required for CRBSI management to avoid loss of the access site. This approach is especially useful in patients awaiting fistula maturation or transplantation. If catheter retention is planned, a longer observation period is needed to look for the recurrence of infection. Urokinase as a thrombolytic agent in the antibiotic lock solution was effective in treating CRBSI. However, some limitations are worth noting. The effective and safe doses and adverse effects of urokinase-based lock therapy need to be investigated in more randomised controlled trials using real-world data. Further research on the stability and compatibility of various antibiotics in combination with urokinase may be beneficial for the expansion and optimisation of antibiotic lock therapy.
